# Exploring Rare Earth Element behavior in the Mount Etna volcanic aquifers (Sicily)

**DOI:** 10.1007/s10653-024-02020-4

**Published:** 2024-06-07

**Authors:** Salvatore Dominech, Cinzia Federico, Lorenzo Brusca, Silvia Fornasaro, Sergio Bellomo, Walter D’Alessandro

**Affiliations:** 1 Istituto Nazionale di Geofisica e Vulcanologia, Sezione di Palermo, Via La Malfa 153, 90146 Palermo, Italy; 2 Dipartimento di Scienze della Terra, Università di Pisa, Via Santa Maria 53, 56126 Pisa, Italy

**Keywords:** Drinking water, Rare Earth Elements, Water–rock interaction

## Abstract

This study presents the first data on REY (Rare Earth Elements plus Yttrium) in the aquifer of Mount Etna (Sicily, Italy). Patterns normalized to chondrites indicate strong water–rock interaction, facilitated by a slightly acidic pH resulting from the dissolution of magma-derived CO_2_. REY patterns provide insights into the processes of both mineral dissolution and the formation of secondary phases. The relative abundance of light to heavy rare earth elements is compatible with the prevailing dissolution of ferromagnesian minerals (e.g., olivine or clinopyroxenes), reinforced by its strong correlation with other proxies of mineral dissolution (e.g., Mg contents). Pronounced negative Ce anomalies and positive Y anomalies demonstrate an oxidizing environment with continuous formation of secondary iron and/or manganese oxides and hydroxides. The Y/Ho fractionation is strongly influenced by metal complexation with bicarbonate complexes, a common process in C-rich waters. In the studied system, the measured REY contents are always below the limits proposed by Sneller et al. (2000, RIVM report, Issue 601,501, p. 66) for surface water and ensure a very low daily intake from drinking water.

## Introduction

The International Union for Pure and Applied Chemistry (IUPAC) defines Rare Earth Elements as a group of 17 elements, including 15 lanthanides (La, Ce, Pr, Nd, Pm, Sm, Eu, Gd, Tb, Dy, Ho, Er, Tm, Yb, Lu) plus Yttrium and Scandium (for this reason, frequently named REY). REY have many applications in electronics and green technologies, such as permanent magnets, batteries, catalysts, displays, wind turbines, electric vehicles, and solar panels (Shin et al., 2019). In recent times, REY have also gained further interest because of their increasing release in the environment due to their use in human activities and products. The mining, refining, and recycling activities amplified the exposure of workers to REY. While an increasing number of studies are reporting on the adverse effects on human beings, regulations on their management are still insufficient (Pagano et al., 2015; Rim et al., 2013; Shin et al., 2019; Waring & Watling, 1990).

A sometimes-undervalued source of REY is represented by active volcano-hydrothermal settings, where hot volcanic fluids can transport REY increasing their mobility in shallow environments (e.g., soil, groundwater) owing to high temperature, high acidity, and reducing conditions. The research studies on REY in currently active hydrothermal systems mainly concern the submarine settings and the nearby sediments in Mid-Ocean ridges, because of the interest in studying fluid-rock interactions and their potential employment in the mining industry (Bau & Dulski, 1999; Douville et al., 1999; Hannington et al., 2005; Klinkhammer et al., 1994; Michard et al., 1983; Takaya et al., 2018; Zheng et al., 2016). Only a few studies deal with investigations on REY in shallow marine settings (Craddock et al., 2010; Falcone et al., 2022; Pichler et al., 1999; Price et al., 2013, 2015) or in estuarine areas (Arienzo et al., 2022). In continental areas, most studies focus on sites polluted by mining activities (Hao et al., 2016; Liu et al., 2022; Tian et al., 2018).

REY’s natural and anthropogenic flows may exert cumulative damaging effects (MacMillan et al., 2017), altering the expected REY distribution (Tepe et al., 2014) and disturbing biogeochemical cycles, especially in natural aquatic systems. Numerous studies observed several ecotoxicological effects of REY on living organisms (Malhotra et al., 2020; Martino et al., 2022; Pagano et al., 2019). For humans, the worst effects are found in dysfunctional neurological disorders, fibrotic tissue injury, pneumoconiosis, and male sterility (Gwenzi et al., 2018).

Despite the chemically coherent properties originating from their trivalent charge and similar ionic radii, some subtle differences existing among REY make them a powerful tool for tracing geochemical processes on Earth. The systematic decrease of the ionic radii across the series with increasing atomic number (which is called the “lanthanide contraction”) and the differences in their electronic configuration are the cause of fractionation processes among individual REY in the aquatic environment (e.g., aqueous complexation, surface complexation, mineral dissolution/precipitation). The REY also display strong sorption characteristics, particularly at high pH, onto mineral surfaces (Erel & Stolper, 1993; Sholkovitz et al., 1994). In solution, trivalent REY, classed as hard ions, form complexes with hard ligands, such as F^−^, SO_4_^--^, CO_3_^--^, PO_4_^---^, and OH^−^ (Brookins, 1989; Wood, 1990). In general, free ions (M^+++^) and sulfate complexes usually predominate at low pH, while carbonate and bicarbonate species are predominant at neutral to basic pH (Kevin et al., 1996; Wood, 1990).

In this study, we present the data on REY measured in groundwater samples collected on Mt. Etna volcano (Italy), with a particular focus on drinking water resources. Indeed, excepting two sites, all sampled waters are used as drinking water by public or private aqueducts, or bottled for sale. Due to the low expected contents in drinking water, we applied an enrichment procedure, based on the attitude of REY to be co-precipitated on solid hydroxides (i.e., Mg(OH)_2_). We show that key factors in controlling the abundance of REY in Etnean groundwater are the water acidity and the carbon contents, as they influence the rock dissolution, and the formation of solution complexes or secondary oxo-hydroxides. The concentration of REY never exceeds the maximum permissible concentrations proposed by Sneller et al. (2000) in shallow waters, based on data on ecotoxicology and environmental chemistry, although maximum acceptable limits for REY in drinking water are not available from any international health organization.

## Study area

Mt. Etna is the largest active volcano in Italy, covering an area of about 1,200 km^2^. It is located on the eastern coast of Sicily, in the metropolitan area of Catania. The volcanic edifice lies over Miocene flysches (to the northwest) and Pleistocene clayey formations (to the southeast), overlying huge carbonate sequences (≈10 km of thickness). The volcanic activity of Mt. Etna volcano started in the Middle Quaternary (0.5 Ma) as a consequence of the African plate margin breaking up during its collision with Europe (Barberi et al., 1974). The volcanism of Etna is characterized by frequent central and lateral eruptions (Tanguy et al., 1997), alternating effusive and explosive activities. Recent volcanic activity produces alkali basalts and hawaiites, with relatively constant compositions (Chester et al., 1985; Correale et al., 2014; Joron & Treuil, 1984; Romano, 1982; Tanguy et al., 1997), and also generates a strong volatile emission through open-conduit degassing processes (Aiuppa et al., 2006; D’Alessandro et al., 1997; Hirn et al., 1997).

**Fig. 1:**
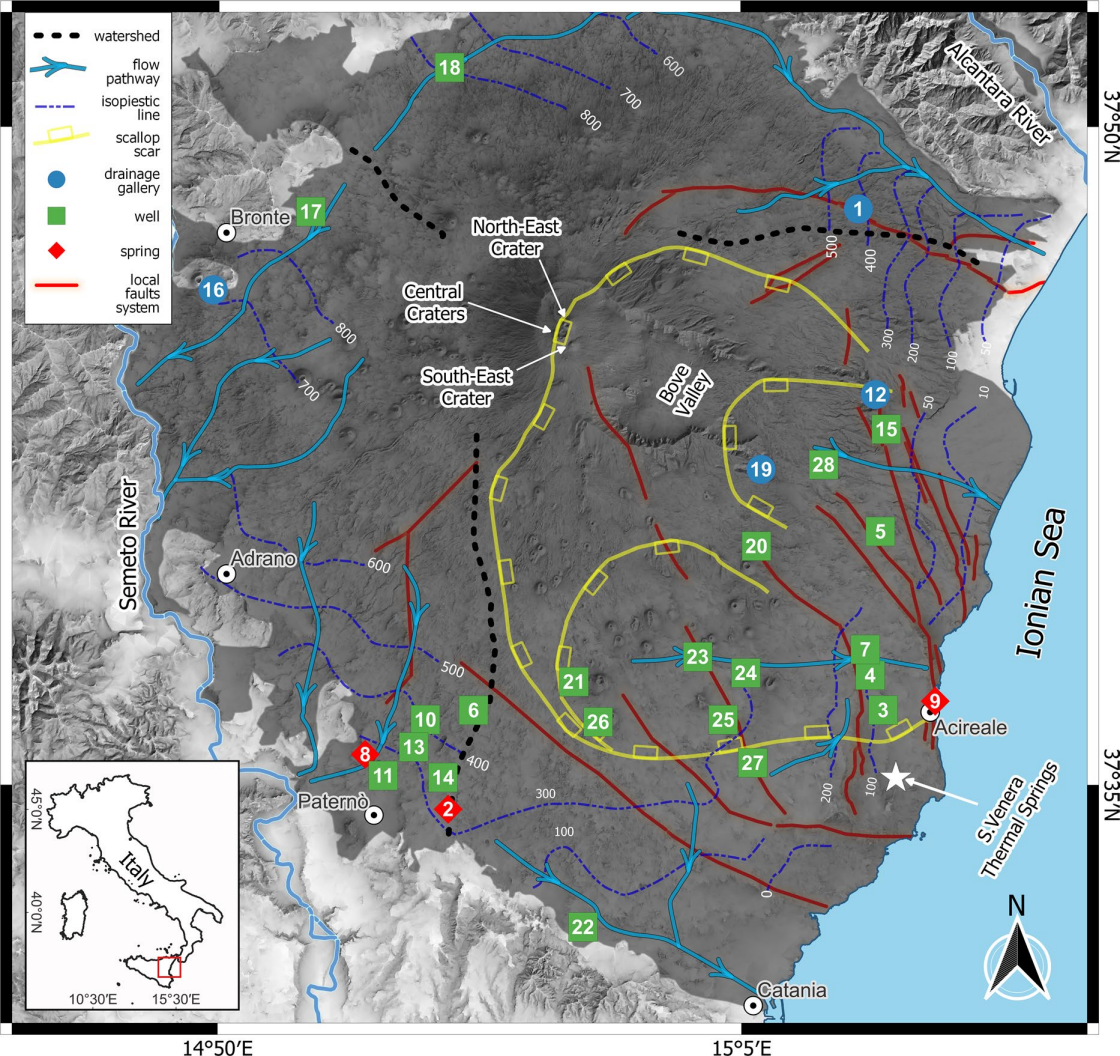
Study area showing the location of the 28 sampling points, classified by their type (drainage gallery, spring and well). The gray shaded area represents the volcanic deposits (lavas and pyroclastics). The map also shows the steep horseshoe-shaped depression in the eastern sector, the watersheds, the major flow pathways of groundwater and isopiestic lines (from Branca & Ferrara, 2013). Local faults systems are also reported from Barreca et al. (2013).The basemap is a TINItaly Digital Elevation Model (Tarquini et al., 2023)

Typical Etnean aquifers can be described as unconfined and hosted by highly permeable volcanites (permeability values ranging from 2.5 × 10^−7^ to 2.9 × 10^−6^ cm^2^) (Ferrara, 1975; Ferrara & Pappalardo, 2008; Schilirò, 1988), overlying a sedimentary basement with an average permeability of 10^−10^ cm^2^ (Bellia et al., 2015). The morphology of the sedimentary basement has total control over the flow pathways of groundwaters (Branca & Ferrara, 2013). Its highest elevation (~ 1300 m a.s.l.) is located beneath the volcano’s northwestern flank. Most springs and wells are located in the southern and eastern flanks. The latter setting is likely due to the basalt layers dipping towards the sea (E-SE), controlling the groundwater flow paths in the same direction (Branca & Ferrara, 2013; Ogniben, 1966). According to the geological and hydrological features, the Etna complex is generally divided into three distinct hydrogeological basins: (a) the northern, feeding the Alcantara River; (b) the western, feeding the Simeto River; (c) the eastern, which flows directly into the Ionian Sea. The eastern sector is characterized by the presence of a wide and deep horseshoe-shaped depression, about 17 km wide and over 20 km in length, with steep slopes along its inner walls (Fig. 1). According to the water-stable isotope data, meteoric water is the predominant recharge source, excluding any possibility of seawater infiltration (D’Alessandro et al., 2004; Liotta et al., 2013).

Groundwaters of the Etnean area have been widely studied in the last decades (Aiuppa et al., 2000, 2003; Brusca et al., 2001; Federico et al., 2017; Liotta et al., 2017) and three main processes have been recognized as factors controlling their variability in terms of chemical composition:

(i) The dissolution of magma-derived CO_2_ has been identified as one of the major causes of the leaching of host basalts (Aiuppa et al., 2000; Brusca et al., 2001; Federico et al., 2017; Liotta et al., 2016). It brings to the groundwater compositions ranging from HCO_3_^-^-Na^+^ to HCO_3_^-^-Ca^++^ or HCO_3_^-^-Mg^++^. These water types are predominant in the fractured areas where magmatic CO_2_ can ascend toward the surface and dissolve into the aquifer (D’Alessandro et al., 1997)**.**
(ii) The mixing with brackish waters hosted within the sedimentary basement significantly contributes to the high concentrations of Na^+^, Cl^-^, B, and Li^+^ (Federico et al., 2017; Liotta et al., 2017). This is particularly evident in the area of Paternò, Adrano, and Bronte (Fig. 1).
(iii) The acidic gas species HCl and SO_2_, carried by the volcanic plume, contribute directly from rainfall and through acidic water-rock interaction, contribute to the relative enrichments in Cl^-^ and SO_4_^--^ (Liotta et al., 2017). This is mainly observed in the Eastern sector, which is fumigated by the volcanic plume due to the prevailing eastward direction of the dominant winds (Calabrese et al., 2015).

The great differences in several parameters in the Etnean aquifers can be easily ascribed to the variety in hydrological characteristics (e.g., flow pathways, residence times, altitude, and amount of meteoric recharge) and the heterogeneity of the CO_2_ paths, which mainly control the water–rock interaction processes (Brusca et al., 2001).

## Materials and methods

### *Sampling and analytics*

The sampling campaign was held in two stages, November and December 2021, leading to the collection of 28 groundwater samples. Groundwaters were collected in wells, springs, and galleries, in the area surrounding Mt. Etna (Fig. 1). Galleries extend from tens to thousands of meters (galleries shorter than 10 m were considered as springs) and often have large diameters (> 2 m). Physicochemical parameters such as temperature, pH, redox potential (as Eh), and electrical conductivity (EC) were measured in the field. At each sampling site, four bottles (subsamples) were collected and treated differently according to the type of chemical analysis to be performed. All the analyses were performed at the laboratories of the INGV-PA. A raw aliquot (50 mL) was collected from the untreated subsample for the estimation of the total alkalinity (Alk_T_) through the Titrator Compact G20S with HCl 0.1 N. Three other subsamples were filtered on-site, using 0.45 μm cellulose acetate membranes (*Cytiva Whatman™*). The subsamples considered for cations and REY analyses were acidified with Ultrapure HNO_3_ (*Carlo Erba®*) for their proper conservation, while the subsample for anion analysis was preserved unacidified. Cations and anions were measured by ionic chromatography (*Dionex ICS-1100*). A 50 mL aliquot, collected from an acidified subsample, was stored in a Polypropylene (hereafter *PP*) vessel, and used for the determination of REY concentrations through a pre-concentration process as described below. 

### *Pre-concentration process: triethylamine-assisted REY co-precipitation*

The REY concentrations in groundwater are usually in the order of ng/L. Due to the expected low concentrations, the samples underwent selective enrichment processes, using the approach of the co-precipitation onto newly forming Mg(OH)_2_ solid phases. The pre-concentration process allowed us to measure ultra-low concentrations (ng/L) starting from a 50 mL sample, enriching REY by a factor of ≈ 7. According to the method proposed by Arslan et al. (2018), the co-precipitation of REY onto the hydroxides is assisted by the triethylamine (*TEA*), an aprotic base, and this produces quantitative scavenging of a large suite of trace elements with oxidation state (III) or higher. Almost all REY present a principal oxidation state of (III), except for Ce (III/IV) and Eu (II/III). Thus, by acidifying samples with HNO_3_ (*Ultrapure- Carlo Erba®*), which acts as an oxidizing agent, we can assume that almost all Eu (II) is oxidized into Eu (III). Since the method proposed by Arslan et al. (2018) was originally developed for seawaters, which are typically richer in Mg^++^ than groundwaters, we implemented some changes to make it suitable for analysis of groundwater. We firstly added MgCl_2_ • 6H_2_O (*magnesium chloride hexahydrate; 99* + *%—ACS, ISO certified*) to the solution to foster the formation of Mg(OH)_2_.

The acidified aliquot of the sample (50 mL) was homogenized in a 1-h ultrasonic bath. We prepared a solution with a measured concentration of Mg of 34,400 mg/L. The Mg-concentrated solution was previously treated with *TEA (Ultrapure)* to remove further impurities. Thus, adding an aliquot of 500 µl into the 50 mL sample, we added 344 mg/L of Mg (dilution factor 1:100). We further added 300 µL of *TEA* to each sample. If hydroxide formation was not clearly visible after about 1 h (i.e., the solution still appeared transparent), especially in samples with an initial low pH, we added an extra aliquot of *TEA* (100 µL) to foster the reaction. The samples were centrifuged at 6,000 rpm for 20 min. The supernatant was collected in a different 50 mL PP vessel, and analyzed later after an additional cycle of enrichment to confirm the efficiency of the phase separation. The precipitate was digested at room temperature in 500 µL of Ultrapure HNO_3_ with subsequent addition of 1 mL of *MilliQ* water (18.2 MΩ). After a gentle shaking of the solution, the concentrated solution was transferred into a 10 mL PP tube to remove the precipitate from the walls of the conical tube. We rinsed the original tube with *MilliQ* water 2–3 times to dissolve eventual sample residues completely, and finally, we added *MilliQ* water to the final volume of 7 mL. The pre-concentrated sample was finally analyzed for the 15 selected elements (La, Ce, Pr, Nd, Sm, Eu, Gd, Tb, Dy, Ho, Er, Tm, Yb, Lu and Y) using an ICP-MS (Agilent 7800) at INGV-PA. The actual concentrations were finally retrieved by correcting the measured values for the enrichment factor (≈ 7), measured dividing the original volume of the sample (50 mL) by the final volume of the concentrated sample (7 mL). In order to verify the purity of the reagents, procedural blank samples underwent the same pre-concentration process. Recovery percentages were estimated by comparing the analytical measurement against the calculated concentrations obtained by diluting a highly concentrated stock solution. The median percentage of recovery of REY was estimated to range between 81 ± 11 and 106 ± 9 in the different samples. The precision of the analysis for all REY was assessed as RSD (Relative Standard Deviation), never exceeding 17%. Recovery percentages apparently higher than 100% are to be ascribed to the uncertainty in the measurement and, however, the values always fall within the analytical error of 17%. The average procedural detection limit has been evaluated at 0.15 ng/L.

We further estimated the analytical accuracy using international water reference material (*SLRS-4*, Lawrence et al., 2006). The median accuracy, expressed as a percentage, for REY was evaluated to be 3.3%.

#### Results

#### Major ions and REY contents

**Fig. 2:**
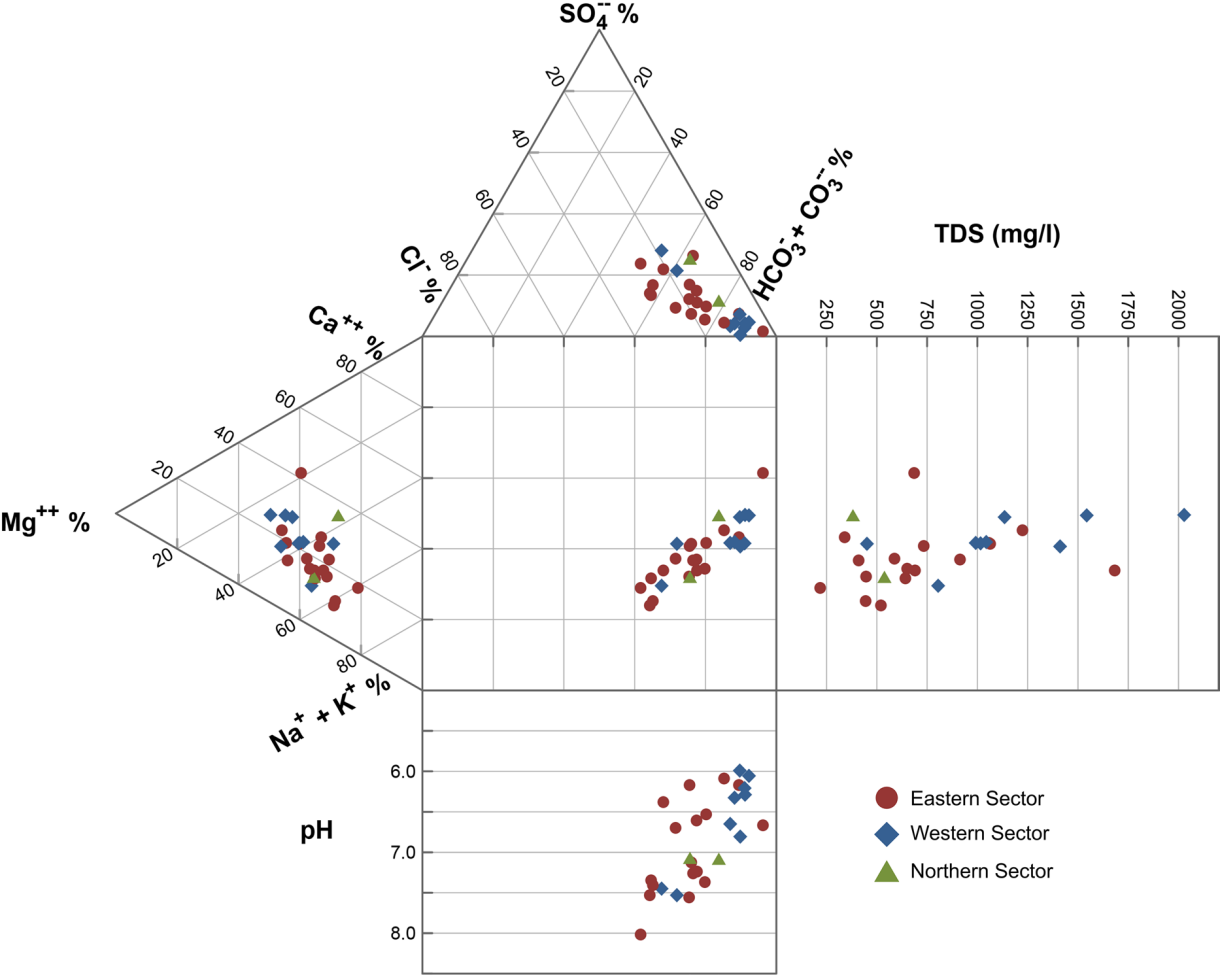
Extended Durov plot showing the concentrations (in %) of the major anions and cations in groundwaters. Samples are classified by hydrogeological sector, according to Branca and Ferrara (2013). Total Dissolved Solids (TDS, in mg/L) and pH are also shown.

The main results of the analysis of the major elements are summarized in Table 1 and plotted in a Durov class plot (Fig. 2).

**Table 1:** Physico-chemical parameters of the groundwater samples, total alkalinity, total dissolved solids (TDS), water types and major ions contents

***ID***	***Location***	***Lat***	***Lon***	***Elevation (a.m.s.l.)***	***Sample type***	***Hydrogeological Sector***	***pH***	***Cond. (µs/cm)***	***Eh (mV)***	***T (°C)***	***Tot Alk. (meq/l)***	***TDS (mg/l)***	***Water type***	***Na***^***+***^*** (mg/l)***	***K***^***+***^*** (mg/l)***	***Mg***^***++***^*** (mg/l)***	***Ca***^***++***^*** (mg/l)***	***F***^***-***^*** (mg/l)***	***Cl***^***-***^*** (mg/l)***	***NO***_***3***_^***-***^*** (mg/l)***	***SO***_***4***_^***- -***^*** (mg/l)***	***Ionic Balance (%)***
**1**	Rocca Campana	37.79898	15.13631	738	gallery	N	7.09	723	255	11.7	4.52	538	HCO_3_^-^-Na^+^	80.2	12.5	32.6	21.2	0.19	30.1	*n.d.*	85.5	-5.2
**2**	Acquarossa	37.57109	14.93998	352	spring	W	6.29	2238	201	18.2	23.9	2029	HCO_3_^-^-Mg^++^	144	14.9	156	129	0.38	63.8	6.82	53.8	4.2
**3**	Ellera	37.60864	15.14778	186	well	E	7.35	1044	154	17.7	4.99	642	HCO_3_^-^-Na^+^	97	14.9	38.4	25.7	0.57	87.2	18.0	55.7	-1.2
**4**	Masaracchio	37.62189	15.14163	256	well	E	7.53	769	175	16.6	3.9	520	HCO_3_^-^-Na^+^	101	11.0	26.7	14.4	0.57	69.1	13.6	46.1	-7.2
**5**	P31	37.67649	15.14657	241	well	E	6.17	992	211	15.5	6.46	733	HCO_3_^-^-Na^+^	84.8	19.9	40.6	47.7	0.38	55.0	11.8	77.8	-0.7
**6**	P5	37.60866	14.95208	611	well	W	5.99	1272	202	14.6	12.8	1136	HCO_3_^-^-Mg^++^	88.7	17.6	74.2	79.8	0.38	35.5	4.96	51.9	4.1
**7**	Ranieri	37.63168	15.13931	293	well	E	7.41	644	165	15.9	3.29	445	HCO_3_^-^-Na^+^	82.8	9.38	21.9	14.0	0.57	54.2	14.3	47.1	-4.8
**8**	Romito	37.59189	14.90035	284	spring	W	6.81	1651	-42	15.4	16.6	1413	HCO_3_^-^-Mg^++^	142	16.4	102	64.1	0.57	65.2	*n.d.*	3.84	2.1
**9**	S.M. La Scala	37.612	15.17268	15	spring	E	6.7	842	192	15.1	5.13	589	HCO_3_^-^-Na^+^	75	13.7	37.7	30.5	0.57	64.9	19.2	34.6	-2.5
**10**	S59	37.6051	14.92907	476	well	W	6.21	1202	164	14.5	11.9	1016	HCO_3_^-^-Mg^++^	99.6	18.4	61.8	53.7	0.57	35.5	3.72	17.3	5.9
**11**	Ardizzone	37.58382	14.90892	281	well	W	6.65	1300	170	n.d.	10.98	991	HCO_3_^-^-Mg^++^	105	17.6	63.7	56.9	0.57	51.4	4.96	21.1	-0.9
**12**	Cavagrande	37.72849	15.1443	294	gallery	E	8.02	340	140	13.1	1.47	220	HCO_3_^-^-Na^+^	38.4	8.21	8.0	11.6	0.19	27.7	2.48	33.6	-3.0
**13**	Pozzo S.Vito	37.59468	14.92349	403	well	W	6.33	1232	150	15.9	11.7	1045	HCO_3_^-^-Mg^++^	110	18.4	63.9	60.9	0.57	46.4	5.58	27.9	0.7
**14**	Raffo 1	37.58312	14.93752	398	well	W	6.06	1680	178	15.9	17.9	1544	HCO_3_^-^-Mg^++^	119	20.3	110	110	0.38	38.3	5.58	45.2	-0.7
**15**	S. Paolo	37.71531	15.14947	228	well	E	6.61	1193	170	15.8	8.54	914	HCO_3_^-^-Na^+^	117	20.3	43.3	51.3	0.19	65.2	8.68	87.4	5.2
**16**	Ciapparazzo	37.76822	14.82733	759	gallery	W	7.45	1141	281	11.1	6.03	806	HCO_3_^-^-Na^+^	121	15.6	47.4	24.9	0.57	73.0	5.58	151	5.2
**17**	Musa	37.79798	14.87406	996	well	W	7.53	625	205	10.3	3.48	450	HCO_3_^-^-Na^+^	54.7	11.7	21.4	32.9	0.57	35.1	22.9	58.6	0.1
**18**	S.Caterina	37.85277	14.94043	893	well	N	7.1	508	188	9.3	3.8	381	HCO_3_^-^-Na^+^	39.1	7.43	17.0	36.1	0.19	18.4	5.58	25.9	-2.2
**19**	S.Giacomo	37.70012	15.08956	751	gallery	E	6.67	779	18	12.1	8.32	686	HCO_3_^-^-Ca^++^	32.7	8.21	41.6	72.5	0.19	9.22	*n.d.*	6.72	0.9
**20**	Ilice	37.67098	15.08735	696	well	E	6.17	429	176	9.1	3.68	340	HCO_3_^-^-Na^+^	35.2	9.78	17.8	23.7	0.38	10.6	3.10	15.4	-1.5
**21**	Piano Elisi	37.6195	15.0001	768	well	E	6.53	1200	118	17	9.64	1058	HCO_3_^-^-Mg^++^	100	23.5	73.9	53.3	0.38	67.4	90.5	59.6	4.7
**22**	Musmeci	37.52634	15.00436	230	well	E	7.13	1348	243	17.1	9.3	1065	HCO_3_^-^-Mg^++^	119	22.3	72.2	63.7	0.38	92.5	81.2	46.1	-0.2
**23**	Pedara	37.62896	15.0592	684	well	E	7.56	622	126	15.5	4.01	447	HCO_3_^-^-Na^+^	66.7	11.7	23.1	20.4	0.57	37.9	8.06	33.6	-2.9
**24**	Muri Antichi	37.6228	15.08219	550	well	E	7.26	552	52	14.3	3.49	410	HCO_3_^-^-Mg^++^	48.5	8.21	30.2	16.8	0.38	20.2	3.10	70.1	-0.7
**25**	Sacro Cuore	37.60506	15.07137	500	well	E	7.37	886	96	16.9	6.61	650	HCO_3_^-^-Na^+^	83.2	13.7	37.4	26.9	0.38	52.8	9.30	23.1	3.9
**26**	Serafica	37.60425	15.01162	653	well	E	6.09	1520	175	18.5	13.5	1224	HCO_3_^-^-Mg^++^	100.7	26.6	88.7	71.3	0.38	72.0	5.58	34.6	2.6
**27**	Etna acque	37.58866	15.08545	374	well	E	7.24	989	164	17.8	6.57	690	HCO_3_^-^-Na^+^	92.7	14.1	38.9	29.7	0.38	54.6	11.2	48	2.6
**28**	Primoti	37.70188	15.11949	503	well	E	6.38	2258	104	19.1	13.2	1682	HCO_3_^-^-Na^+^	244	40.7	91.4	82.6	0.76	171	4.96	242	-0.4

The samples were grouped by sectors (East, West, North, Fig. 1), which represents the main hydrogeological structures, bounded by the watershed identified by Branca and Ferrara (2013). As observed in the Durov plot of Fig. 2, the main hydrogeochemical facies of Etnean groundwaters are HCO_3_^−^-Na^+^ and HCO_3_^−^-Mg^++^, with the only exception of sample #19, classified as HCO_3_^−^-Ca^++^. Total Dissolved Solid (TDS, major ions contents plus total alkalinity) ranges from 220 mg/L in sample #12 to 2,030 mg/L in sample #2. All water samples collected in the southernmost area of the western sector belong to the HCO_3_^−^-Mg^++^ class, whereas those collected in the eastern flanks of the volcano are more enriched in Na^+^. The groundwaters preserving the magnesian character are also identified as the most acidic in this sector. Further, the western samples are in general more acidic than the eastern ones, characterized by an average pH < 7.

The concentrations of REY in the analyzed samples are listed in Table 2, along with REY concentrations in blank samples, the median RSDs and the recovery percentages.

**Table 2:** REY concentrations for the 24 groundwater samples (in ng/L). The median recovery rate and the median RSD for a single element are also shown. In bold are the samples that presented reliable values along all the REY series

***ID***	***La***	***Ce***	***Pr***	***Nd***	***Sm***	***Eu***	***Gd***	***Tb***	***Dy***	***Y***	***Ho***	***Er***	***Tm***	***Yb***	***Lu***	*∑****REY***
**2**	**22.3**	**14.6**	**4.18**	**18.4**	**3.38**	**1.11**	**6.15**	**0.98**	**7.78**	**156**	**2.07**	**7.68**	**1.24**	**8.25**	**1.55**	**255**
3	3.44	2.55	0.39	2.02	0.24	<0.15	1.75	<0.15	0.47	5.03	<0.15	0.46	<0.15	0.64	<0.15	17
4	0.68	<0.15	<0.15	0.61	<0.15	<0.15	0.56	<0.15	0.17	1.97	<0.15	<0.15	<0.15	<0.15	<0.15	4
**5**	**86.7**	**17.3**	**9.35**	**38**	**5.48**	**1.44**	**8.13**	**1.16**	**8.44**	**134**	**2.51**	**8**	**1.33**	**10.5**	**2.21**	**334**
**6**	**38.4**	**8.62**	**4.96**	**21**	**3.88**	**1.18**	**5.75**	**0.81**	**6.24**	**100**	**1.82**	**6.25**	**0.91**	**6.4**	**1.11**	**207**
7	1.77	1.48	0.23	1.1	0.34	<0.15	0.78	<0.15	<0.15	2.65	<0.15	0.17	<0.15	0.18	<0.15	9
**8**	**10.2**	**14.4**	**1.74**	**8.19**	**1.36**	**0.49**	**1.93**	**0.29**	**2.46**	**44.6**	**0.96**	**2.93**	**0.49**	**3.71**	**0.61**	**94**
9	4.99	7.21	0.94	4.05	0.69	0.22	1.02	<0.15	0.69	6.19	<0.15	0.45	<0.15	0.76	0.2	27
**10**	**16**	**4.55**	**3.82**	**19.3**	**4.44**	**1.39**	**6.52**	**1.08**	**7.6**	**98.9**	**2.16**	**6.5**	**1.02**	**6.83**	**1.29**	**181**
**11**	**18.5**	**2.41**	**2.72**	**12**	**2.28**	**0.65**	**3.54**	**0.43**	**3.78**	**67.4**	**1.21**	**3.45**	**0.61**	**4.15**	**0.72**	**124**
**13**	**27.6**	**1.38**	**3.73**	**18**	**2.88**	**1.19**	**5.57**	**0.79**	**4.66**	**91.4**	**1.19**	**4.5**	**0.64**	**4.78**	**0.87**	**169**
**14**	**40**	**11.5**	**4.74**	**18.9**	**2.87**	**0.92**	**4.96**	**0.76**	**6.11**	**99.3**	**1.75**	**5.58**	**0.82**	**6.42**	**1.17**	**206**
**15**	**26.9**	**2.44**	**3.48**	**13.7**	**2.16**	**0.82**	**2.68**	**0.41**	**2.88**	**36.2**	**0.89**	**2.4**	**0.38**	**2.98**	**0.62**	**99**
16	3.19	0.69	0.53	2.40	0.37	<0.15	0.51	<0.15	0.48	7.29	0.19	0.5	<0.15	0.84	0.16	17
**17**	**13.5**	**10.7**	**3.03**	**12.4**	**2.85**	**0.68**	**2.99**	**0.34**	**2.25**	**16**	**0.67**	**1.44**	**0.22**	**1.78**	**0.4**	**69**
18	6.42	0.63	0.73	3.65	0.25	<0.15	0.61	<0.15	0.52	9.05	0.28	0.46	<0.15	0.44	<0.15	23
19	2.77	3.71	0.37	1.75	0.16	0.17	0.27	<0.15	0.43	10	0.28	0.29	<0.15	0.53	<0.15	21
**20**	**25.5**	**2.56**	**3.50**	**15.6**	**2.54**	**0.69**	**4.04**	**0.58**	**3.03**	**48.3**	**0.87**	**3.07**	**0.39**	**2.74**	**0.47**	**114**
**21**	**10.2**	**6.06**	**1.38**	**7.3**	**0.96**	**0.48**	**2.11**	**0.32**	**2.53**	**46**	**0.75**	**2.86**	**0.46**	**3.68**	**0.47**	**86**
22	4.88	0.86	0.73	3.96	0.56	0.35	4.18	<0.15	0.98	13.1	0.47	0.93	<0.15	1.59	0.35	33
25	1.09	1.36	<0.15	0.77	0.18	<0.15	0.23	<0.15	0.18	2.41	0.18	<0.15	<0.15	0.28	<0.15	7
**26**	**29.2**	**2.59**	**3.91**	**18.8**	**3.44**	**1.18**	**4.84**	**0.67**	**5.2**	**94.1**	**1.63**	**5.5**	**0.76**	**5.59**	**0.98**	**178**
27	1.07	0.55	<0.15	0.87	<0.15	<0.15	0.38	<0.15	0.21	2.28	<0.15	<0.15	<0.15	<0.15	<0.15	5
**28**	**30.2**	**4.42**	**3.39**	**14.5**	**2.25**	**0.58**	**3**	**0.43**	**3.95**	**57.6**	**1.24**	**3.68**	**0.56**	**4.66**	**0.87**	**131**
*MIN*	0.68	0.55	0.23	0.61	0.16	0.17	0.23	0.29	0.17	1.97	0.18	0.17	0.22	0.18	0.16	4
*MAX*	86.7	17.3	9.4	38	5.5	1.4	8.1	1.2	8.4	156	2.5	8	1.3	10.5	2.2	334
*MEDIAN*	11.9	2.59	3.03	10.1	2.2	0.69	2.84	0.62	2.53	40.4	0.96	2.93	0.62	3.33	0.72	90
*BLANK (avg.)*	0.42	0.48	0.06	0.21	0.09	0.02	0.05	0.01	0.05	0.29	0.02	0.04	0.01	0.02	0.01	
*MEDIAN RECOV. (%)*	102±3	101±7	106±9	98±10	82±13	81±12	104±11	101±13	89±9	104±5	82±12	98±10	101±11	103±8	82±14	
*MEDIAN RSD (%)*	3.45	7.25	8.8	10	15.6	15.3	11.1	13.3	10.4	4.85	15.3	10.8	10.7	7.45	17.2	

The overall quality of the measurement for 15 REY (La, Ce, Pr, Nd, Sm, Eu, Gd, Tb, Dy, Y, Ho, Er, Tm, Yb, Lu) was evaluated acceptable (RSD < 20%) for 24 samples out of 28. From this list, we excluded the data of samples #1, #12, #23 and #24, because they showed concentrations very similar to the procedural sensitivity.

**Fig. 3:**
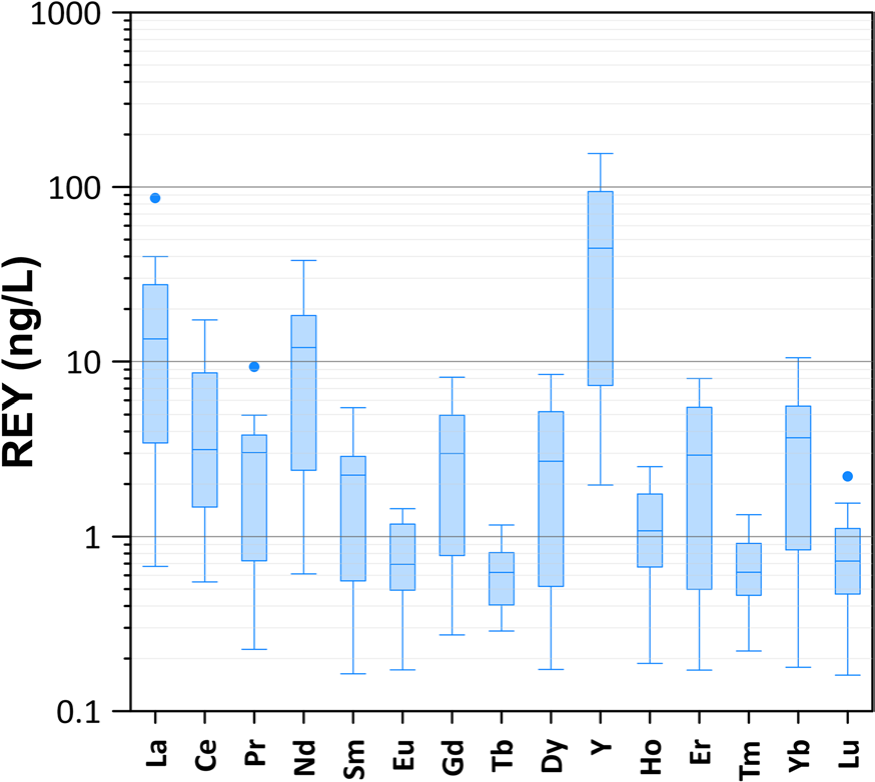
Box plot showing the REY contents in the analyzed water samples

The REY total contents in the different samples display a wide range, spanning from 4 ng/L in sample #4, to 334 ng/L in sample #5. Along the series, Y shows the highest contents, reaching 156 ng/L in sample #2 (Fig. 3). In addition, La and Nd show a wide range with median values 11.9 and 10.1 ng/L, respectively. For La, a local outlier (87 ng/L) is measured in sample #5. The lowest median values (< 1 ng/L) are recorded for Eu, Tb, Ho, Tm and Lu.

**Fig. 4:**
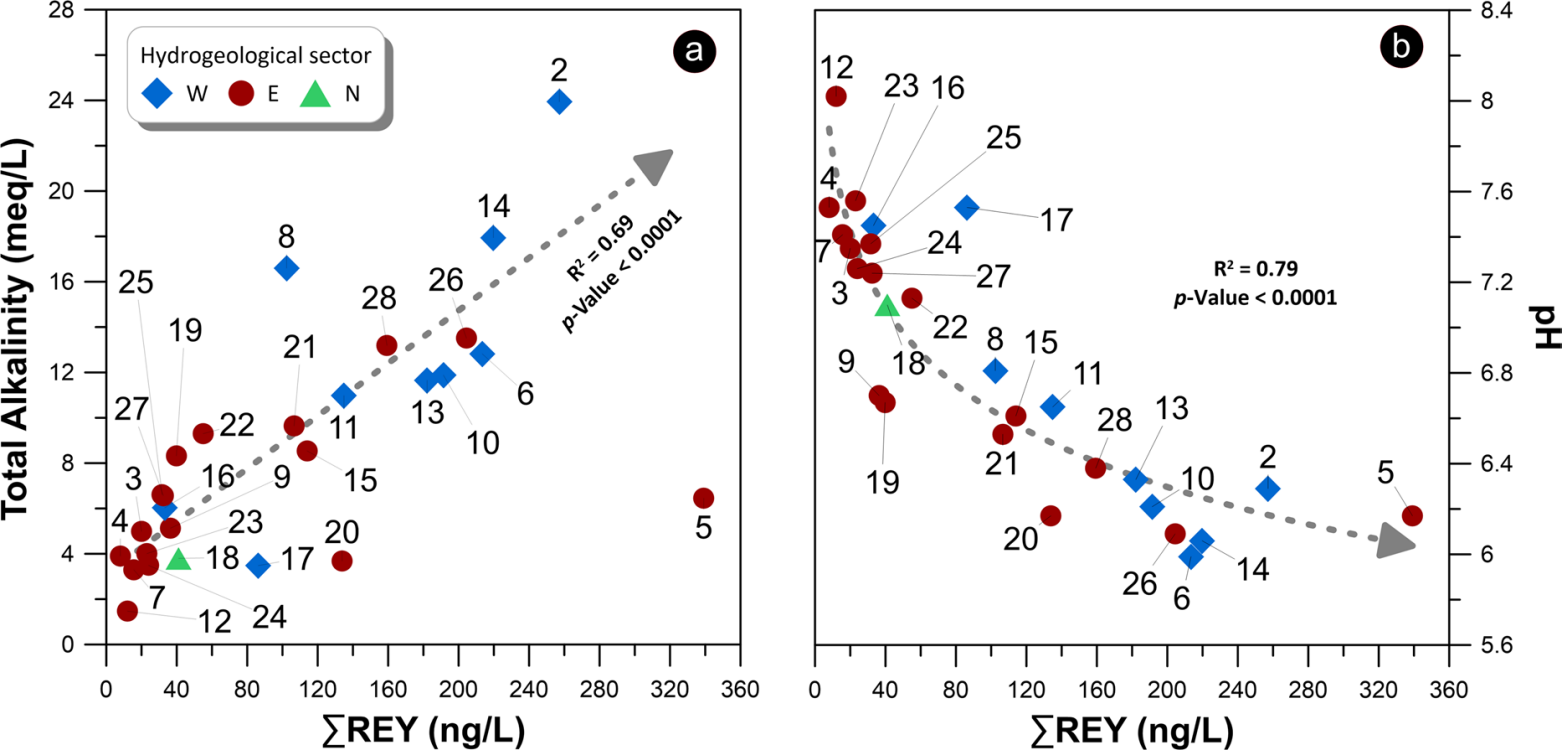
REY content versus total alkalinity **a** and pH **b**. Samples are classified according to the hydrogeological sector they belong to (Branca & Ferrara, 2013). In **a**, samples #5 has been considered as an outlier and then it has been excluded from the regression

As displayed in Fig. 4A, the REY contents are linearly correlated (R^2^ = 0.69) with the total alkalinity, with the only exception of sample #5, which represents an outlier for the La content, the metal that mainly controls the total REY contents. An inverse correlation is observed in Fig. 4B between total REY contents and the pH values of groundwater samples, regardless of the hydrogeological sector they belong to.

##### *REY patterns*

**Fig. 5:**
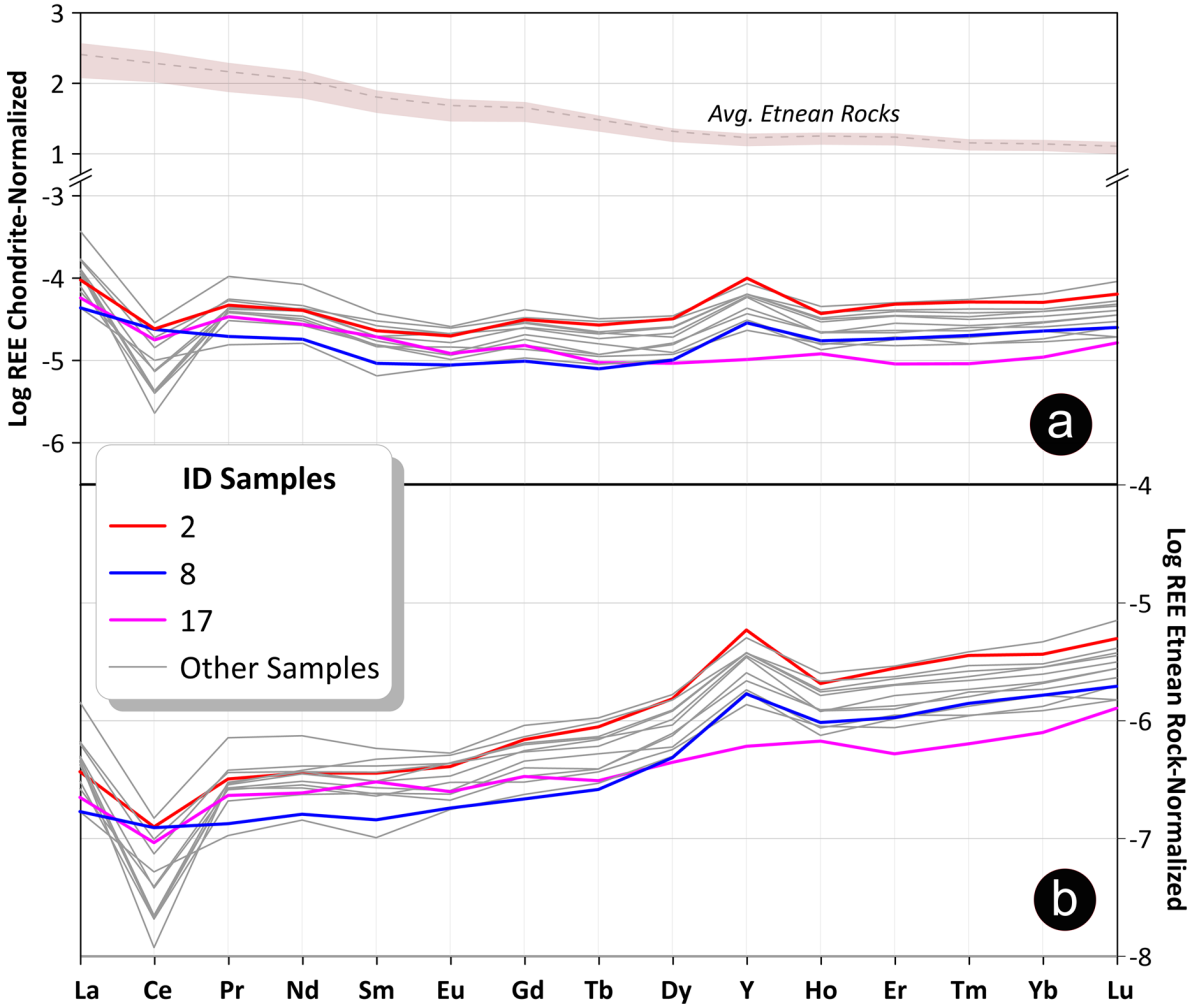
(a) Chondrite-C1 Normalized Patterns for 15 REY (14 lanthanides + Yttrium) in the analyzed groundwater samples. The gray main trend of the Etnean groundwaters is well represented by sample #2 (as the most representative sample) and Other Samples. The Etnean source rocks values were estimated as range (pink shaded area) and average (gray dashed line) of several types of Etnean products like alkaline lavas and tephra (from Correale et al., 2014). (b) Etnean Rock Normalized Patterns for 15 REY (14 lanthanides + Yttrium) in the analyzed groundwater samples. Rock data are from Correale et al., 2014

The REY raw concentrations were normalized to the *Chondrite-C1* values (Anders & Grevesse, 1989), and their patterns are shown in Fig. 5A. Only samples that exhibit a full 15-elements series (the ones in bold in Table 2), are represented. The choice of chondrite normalization is based on the volcanic nature of the Etnean subsoil. Indeed, mafic and ultra-mafic rocks are usually normalized to the reference chondritic concentrations rather than other standards (e.g., Post-Archean Australian Shales or Upper Continental Crust), since the former reflect bulk Earth abundances with higher affinity to primitive magmas. Once normalized, enrichments or depletions of each element compared to the neighbors can be identified as anomalies. The average Etnean rock values were estimated from REY concentrations of different source rocks (alkaline lavas and tephra) reported by Correale et al. (2014).

In Fig. 5A, most samples exhibit a relatively uniform pattern from La to Lu, with pronounced negative anomalies in Ce and positive anomalies in Y. Sample #2, which showed among the highest concentrations with the lowest RSDs, was identified as the most representative sample of the main trend of the Etnean groundwaters (red lines in Fig. 5). Some samples were represented individually due to their different behaviors. Specifically, sample #8 did not show Ce anomaly (blue lines in Fig. 5) and sample #17 displayed normalized Ho concentrations higher than Y (pink lines in Fig. 5). “Other Samples” (marked as gray lines) exhibit fairly homogeneous trends. To highlight processes concerning groundwaters during their interaction with the bedrocks, the REY contents are also normalized to the average Etnean rocks (Correale et al., 2014) (Fig. 5B). The normalized patterns show an almost flat pattern from La to Eu, except for a strong negative Ce anomaly, and an increasing trend from Gd to Lu, with an evident positive Y anomaly.

##### *Quantification of REY anomalies*

Detection of anomalies is based on the presence of individual elements that are higher (or lower) than the corresponding normalized patterns. REY anomalies were quantified calculating a predictable concentration (e.g., REY*), which is generally obtained by interpolating the concentrations of their neighboring REY. As a Ce anomaly could also derive from a possible La enrichment, we discriminated the true Ce anomaly in the analyzed samples by comparing the anomalies of Pr (Pr/Pr*) and Ce (Ce/Ce*)_,_ following the approach proposed by Bau and Dulski (1996)**:**

(Ce/Ce*)_SN_ = Ce_SN_ /(0.5La _SN_ + 0.5Pr_SN_);

(Pr/Pr*)_SN_ = Pr_SN_/(0.5Ce_SN_ + 0.5Nd_SN_);

where Ce* and Pr* are the predictable concentrations and SN stands for shale-normalized values. As for shale reference values, we used the Post-Archean Australian Shales (PAAS) assessed by McLennan (1989).

**Fig. 6:**
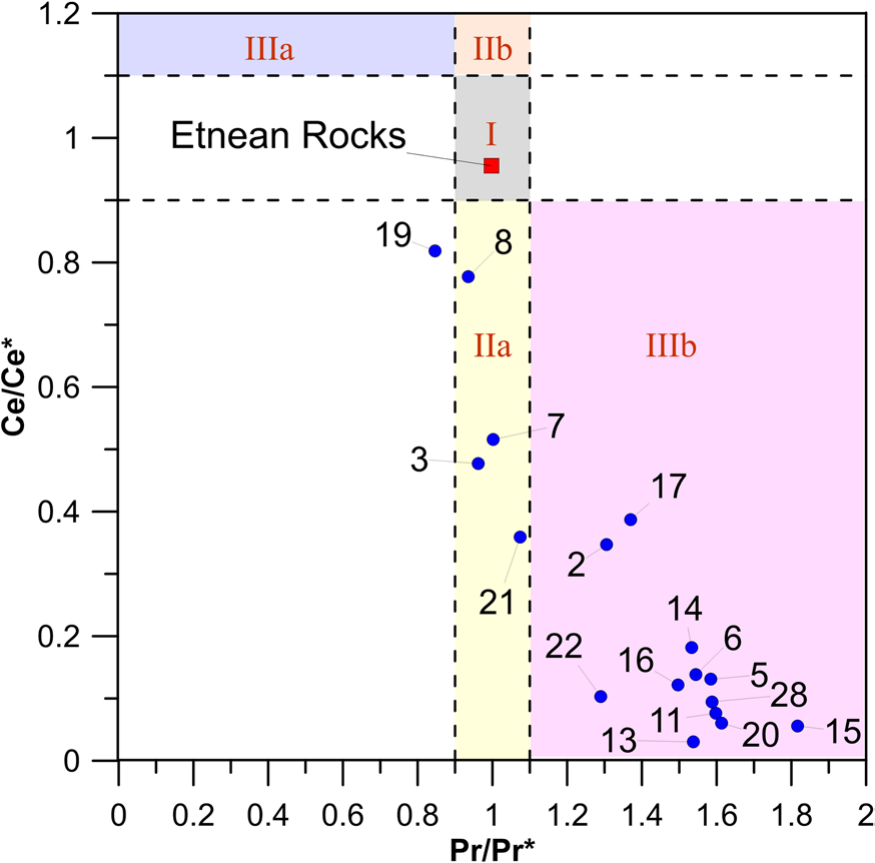
(Ce/Ce*)_SN_ vs (Pr/Pr*)_SN_ graph. Field I: neither Ce_SN_ nor La_SN_ anomaly; field IIa: positive La_SN_ anomaly, no Ce_SN_ anomaly; field IIb: negative La_SN_ anomaly, no Ce_SN_ anomaly; field IIIa: positive Ce_SN_ anomaly; field IIIb: negative Ce_SN_ anomaly. SN stands for shale-normalized (PAAS, McLennan, 1989). For further explanation, see text

In the binary plot of Fig. 6, the “real” negative Ce anomalies are represented by positive Pr/Pr* and negative Ce/Ce* values (field *IIIb*). Most water samples fall in the field of negative Ce anomalies. Only a few samples fall in the field *IIa* (namely sample #19, #8, #3, #7 and #21) which could be related to a positive La anomaly, able to generate false Ce negative anomalies in normalized patterns. We also estimated Gd anomalies, normalized to chondritic reference values, according to the formula proposed by Möller et al. (2021):

(Gd/Gd*)_CN_ = log Gd _CN_ + log Ho _CN_ − (log Tb _CN_ + log Dy _CN_);

where Gd* is the predictable concentration of Gd and CN stands for chondrite-normalized values. Reference values for *Chondrite-C1* refers to the one proposed by Anders and Grevesse (1989).

Calculated anomalies, together with Nd/Yb and Y/Ho chondrite-normalized ratios, are reported in Table 3.

**Table 3:** Calculated REY anomalies. Ce and Pr anomalies are estimated according to the approach proposed by Bau and Dusky (1996). Gd anomalies are estimated according to Moller et al. (2021). Nd/Yb and Y/Ho chondrite-normalized ratios are also reported. SN and CN stand for shale- and chondrite-normalized values, respectively (McLennan, 1989; Anders and Grevesse (1989)

***ID***	***Y/Ho***_***CN***_	(Ce/Ce*)_***SN***_	(Pr/Pr*)_***SN***_	(Gd/Gd*)_***CN***_	***Nd/Yb***_***CN***_
**2**	2.68	0.35	1.31	1.35	0.8
**3**	-	0.48	0.96	-	1.14
**5**	1.89	0.13	1.58	1.68	1.3
**6**	1.96	0.14	1.54	1.68	1.18
**7**	-	0.52	1	-	2.22
**8**	1.65	0.78	0.94	2.12	0.79
**9**	-	0.76	1.01	-	1.91
**10**	1.63	0.13	1.38	1.38	1.02
**11**	1.98	0.08	1.6	2.13	1.04
**13**	2.73	0.03	1.54	1.46	1.35
**14**	2.02	0.18	1.53	1.51	1.06
**15**	1.44	0.06	1.82	1.65	1.65
**16**	1.38	0.12	1.5	-	1.03
**17**	0.85	0.39	1.37	2.11	2.5
**18**	1.15	0.06	1.42	-	2.96
**19**	1.28	0.82	0.85	-	1.19
**20**	1.99	0.06	1.61	1.61	2.05
**21**	2.19	0.36	1.07	1.55	0.71
**22**	1	0.1	1.29	-	0.89
**25**	0.46	1.38	-	-	1.24
**26**	2.05	0.05	1.51	1.84	1.21
**27**	-	0.5	-	-	-
**28**	1.66	0.09	1.59	1.76	1.12

## Discussion

The REY total contents measured in the Etnean aquifer show a wide range of values, ranging from < 5 to 334 ng/L. The samples collected in some sites display very low REY contents, in most cases below 0.15 ng/L. Other water samples, bottled for sale or distributed in private or public aqueducts, have La, Ce, Nd and Y contents as high as tenths or hundreds ng/L. In order to provide some insights on the amount of REY ingested by the population living in the Etna’s area, we tentatively estimate the daily intake related just to the drinking water. Considering the maximum total REY contents (334 ng/L), as measured in sample #5, the estimated daily intake of REY derived from drinking water (assuming a consumption rate of 2 L of water per day) would be about 0.7 µg/d. This value is sensibly lower than the safe daily intake for rare earth elements (100–110 µg/kg/d) evaluated by Zhu et al. (1997). Nevertheless, the effect of bioaccumulation for long-term intake of even small doses should not be neglected and, additionally, in the computation, the intake of REY related to the food preparation, or the irrigation of vegetables should be also considered. Concerning the possible source of REY in the Etnean aquifer, in the following we intend to verify their hypothetical release through the rock leaching, as are most of the other chemicals, according to the available literature studies, and the eventual removal from the aqueous solutions. REY anomalies and fractionation have frequently been used to evaluate water–rock interaction processes, based on the different chemical and physical properties of these elements along the series (e.g., Byrne & Kim, 1990; Censi et al., 2017; Davranche et al., 2017; Falcone et al., 2022). In particular, the fractionation along the series has been demonstrated to be basically controlled by their different affinity in forming surface and dissolved complexes (Byrne & Li, 1995; Lee & Byrne, 1992).

The groundwater samples from the western sector, compared to the other sectors, are generally characterized by higher HCO_3_^−^ contents (Fig. 2) which, paralleled by the lower pH of these waters, indicate the leaching of the host basalts, driven by magma-derived CO_2_, as a dominant factor in this sector (Aiuppa et al., 2003; Brusca et al., 2001; Liotta et al., 2016). Almost all water samples collected in the eastern sectors belong to the HCO_3_^−^-Na^+^ class, and they are more alkaline than those collected in the western sector, with an average pH > 7. TDS values in these samples are lower than 1,000 mg/L. Even in this sector, the origin of HCO_3_^−^ contents is likely related to the dissolution of magma-derived CO_2_. These waters are slightly enriched in Cl^−^, SO_4_^−−^ and Na^+^, and this may suggest a contribution from rising brines, whereas a significant involvement of seawater can be excluded (Federico et al., 2017; Liotta et al., 2017). The waters most enriched in these ions (namely #3, #4 and #7) were all collected from wells very close to one of the surface expressions of the sedimentary brines (S. Venera thermal spring – Brusca et al., 2001), in an area where the impermeable substrate is at a higher altitude with respect to seawater level. Moreover, as deduced by Brusca et. (2001) and Aiuppa et al. (2003), based on a larger dataset compared to the one discussed in the present paper and, namely, on the B/Cl and K/Na values, the direct influence of seawater can be excluded for almost all samples, being evident either the effect of water–rock interaction or the contamination by brines.

It is evident in Fig. 5A that the REY patterns in groundwaters look very different from those in the bedrocks (Correale et al., 2014), and this suggests that they are fractionated during fluid-rock interaction. Indeed, their fluid/rock partitioning coefficients in aqueous systems are generally low and the isomolar leaching, which should characterize the first and immature steps of the process in acidic/hyperacidic conditions (generally associated with hydrothermal and/or volcanic environments) (Atwood, 2012; Inguaggiato et al., 2020; Ogawa et al., 2021), is expected to generate patterns similar to the source rock. Undoubtedly, the leaching of the bedrock is the main source of REY in the Etnean aquifer, as suggested by the relationships of total REY contents with total alkalinity and pH, shown in Fig. 4. Indeed, both total alkalinity and pH can be considered as markers of the acidic leaching of the host-rocks driven by the CO_2_ dissolution, as already suggested in previous studies (Aiuppa et al., 2000; Brusca et al., 2001; Federico et al., 2017; Liotta et al., 2016**)**. Nevertheless, we must consider secondary processes and possible sinks for REY, able to produce the observed patterns and anomalies. In Fig. 3, it is evident that the chondrite normalized patterns of analyzed samples are characterized by (i) a progressive enrichment in HREE compared to LREE, (ii) high Y/Ho ratios in almost all samples, (iii) a marked Ce negative anomaly, (iv) and a slight positive Gd anomaly.

**Fig. 7:**
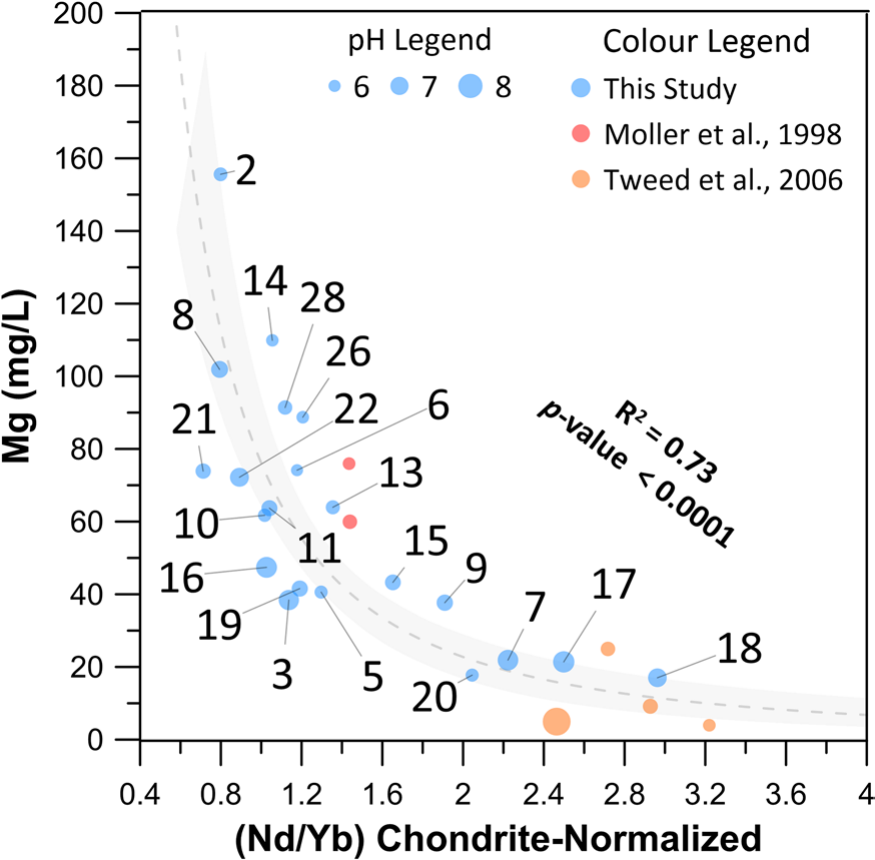
Mg content (in mg/L) vs the Nd/Yb chondrite-normalized values. Blue symbols refer to the samples from this study. Red symbols are waters from alkali basalts (Möller et al., 1998). Orange symbols refer to groundwaters hosted in basalts (Tweed et al., 2006). Bubble sizes are defined according to the pH. The regression is a power law with a confidence interval (gray-shaded area) set at 95%

**Fig. 8:**
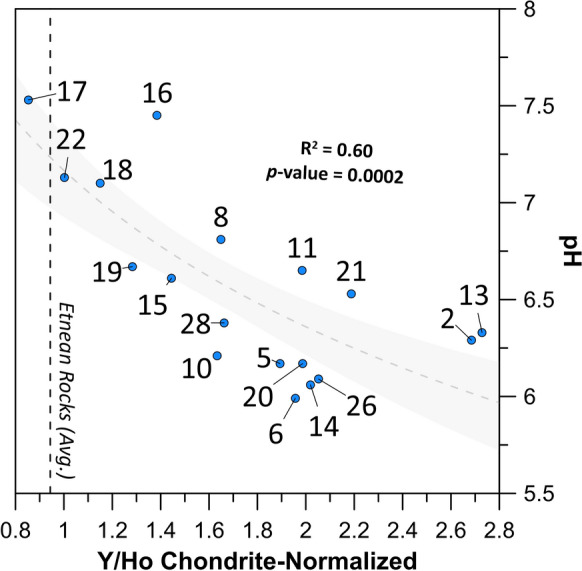
Y/Ho chondrite-normalized values vs pH. The regression is logarithmic with a confidence interval (gray-shaded area) set at 95%. The Etnean rock values were estimated as the average (dashed line) of several types of Etnean products like alkaline lavas and tephra (Correale et al., 2014)

Concerning the bullet point (i), the Nd/Yb ratio are considered as proxies for light/heavy REE fractionation, and their values in the groundwater of Mt. Etna are plotted versus the Mg contents in Fig. 7. To verify if the observed behavior has a general validity, we report in Fig. 7 some literature data on waters, supposed to have interacted with mafic rocks. Möller et al. (1998) reported REY data behavior in mineral waters from NW-Bohemia (Czech Republic) hosted in different volcanic lithologies, such as granites (variably weathered) and alkali basalts. Tweed et al. (2006) also reported the REY concentrations measured in groundwaters from Dandenong Ranges (southeast Australia), hosted in both basaltic and sedimentary aquifers. As observed in Fig. 7 the Nd/Yb ratios values increase as the Mg contents decrease and the pH increases. The Mg content, which is the dominant cation, in particular in groundwater from the western sector of Mt. Etna, is supposed to be directly controlled by the rock leaching (Aiuppa et al., 2000; Brusca et al., 2001). The effect of leaching in the immature stages of weathering, as the relatively low pH values would indicate, is stronger for elements enriched in those minerals with a higher attitude to weathering, such as olivines and pyroxenes (Stefansson, 2001). The Mg-rich composition of olivines and pyroxenes in Etnean bedrock (Correale et al., 2014; Viccaro et al., 2015) would explain the higher Mg contents in the most acidic waters, i.e., those characterized by a weathering process in its early and immature stages. Additionally, olivine and pyroxene, in the volcanics of  Mt. Etna, host preferentially HREE, which are more compatible and favorably incorporated into the crystal lattice than LREE (D’Orazio et al., 1998; Stead et al., 2017). Therefore, the preferential leaching of olivine and pyroxenes would produce an enrichment in HREE in the leaching solution in the first and more acidic steps of the weathering process and, as a consequence, could contribute to a decrease in the Nd/Yb ratio. These inferences also hold true in other geological contexts, wherever groundwaters interact with mafic and olivine-bearing volcanic rocks.

(ii) The chondrite-normalized Y/Ho (Y/Ho_CN_) values (Fig. 8) show an inverse correlation with the pH values (R^2^ = 0.60 with a p-value = 0.002), wherein the samples showing the more acidic behavior (pH < 7) display higher Y/Ho_CN_. In weathering experiments, the Y/Ho ratios turned out as a tracer of the weathering intensity, due to their behavior independent of the “charge and radius” pair (i.e., non-CHARAC, Bau et al., 1996).

Although sharing the same charge and similar radius, Y and Ho differ in their electronic configuration, characterized by empty ([Kr]4d^0^), and partly filled ([Xe]4f^10^) 4f orbital for Y^+++^ and Ho^+++^, respectively. Because the 4f electrons shield the nuclear charge less effectively than electrons in other orbitals, Y has a lower ionization potential and, therefore, a lower attitude to form covalent bonds than its neighbors and, specifically, Ho. In particular, Y–Ho fractionations are apparent during inner-sphere complexation, typically characterized by covalent bonds, with both Fe-oxyhydroxide surfaces (Ohta & Kawabe, 2000), where Y adsorption is limited, and solution ligands, which preferentially bond to Ho than Y (Bau, 1999; Liu & Byrne, 1995; Ohta & Kawabe, 2001; Quinn et al., 2004). Field and laboratory experiments have shown that different stages of basalt weathering produce a wide range of Y/Ho ratios, with a stronger depletion of Y in soil profiles compared to the protolyte in correspondence with the most acidic and intensely weathered soils (Babechuk et al., 2015; Thompson et al., 2013). This would suggest a strong partition of Y in the weathering solution. The Fe-oxyhydroxides, involved in the process of basalt weathering and soil lateritization, are considered the most important phase controlling the fractionation of Y from Ho, mostly occurring at intermediate pH ranges (Thompson et al., 2013). The pH decrease and the parallel increase of solubility of hydroxide species lead to Y/Ho fractionation, driven by the major affinity of Y with the aqueous phase (Tanaka & Kawabe, 2006; Tanaka et al., 2008). This holds true also in the studied samples. Indeed, in the Etnean groundwaters, the increase of Ho concentrations, related to the intense leaching, is paralleled by the increase of the Y/Ho_CN_. The samples with the highest concentration of Ho (e.g., samples #2, #5 and#10) are the samples that even doubled or tripled the bedrock’s Y/Ho_CN_. This suggests a greater enrichment of Y over Ho in the case of intense leaching and release of chemicals to leaching solutions, driven by the higher water acidity. At higher pH values, Ho forms stronger aqueous carbonate complexes than does Y, and this favors the stability of Ho in solution thus decreasing the Y/Ho fractionation (Liu & Byrne, 1995; Quinn et al., 2006).

Concerning Ce(III), most samples show a negative anomaly, which suggests its scavenging on particles in oxidizing conditions (Fig. 6). Oxidizing environments promote the transition from Ce(III) to Ce(IV), making it highly reactive with oxygen to form oxides. Furthermore, the formation of hydrous iron oxides (HFO) and hydrous manganese oxides (HMO) develop the catalytic oxidation of Ce on particles, and very strong negative Ce anomalies in oxygen-rich waters (Möller et al., 2021). For two water samples (i.e., sample #8 and #19), the reducing conditions inhibit the oxidative scavenging of Ce. In the other samples (i.e., sample #3, #7 and #21), only a positive La anomaly is observed, and no Ce anomaly. The positive La anomaly generally observed in studied groundwaters resides on the high stability of La in solution, which shares with Y, discussed above, Gd and Lu a peculiar electronic configuration, characterized by empty, half-filled, and full 4-f orbital, differently to their neighborhoods (Bau et al., 1995; De Baar et al., 1991; Lee & Byrne, 1992). We can hypothesize that some scavenging processes can occur also in the groundwater collected in sample #3, #7 and #21, able to generate a La anomaly, without the significant involvement of Mn-Fe-oxyhydroxides, which otherwise would produce a Ce anomaly.

(iv) The slight Gd anomaly, observed in almost all samples, confirms the widespread occurrence of scavenging processes.

## Conclusions

The REY concentrations in analyzed waters, hosted in the Etna volcanic aquifer, most of them used for drinking purposes, were in the range of 0.16 to 155 ng/L. These contents imply a daily intake far below the safe amount evaluated by Zhu et al. (1997) and are always below the limits proposed by Sneller et al. (2000) although, at present, no sufficient data are available about the negative effects of REY on human health and, in particular, for long-term low-dose exposure.

The normalized REY patterns in the Etnean groundwaters allow us to characterize the processes of water–rock interaction and, in particular, on the extent of leaching and the effect of complexation and removal on alteration minerals, leading to the observed chemical compositions. Given the low pH values measured in many samples, controlled by the input of volcanic CO_2_ mostly in some areas, the leaching of the bedrock appears as the leading process. Indeed, in the most acidic and immature waters, the major ion composition and the REY patterns are compatible with the prevailing leaching of olivine and pyroxene, which are the minerals that have a high attitude to dissolve. At the same time, the Y/Ho ratios increase along with the leaching intensity, given the higher mobility of Y in solution compared to its neighbors. As the pH values increase, the Y/Ho fractionation is reduced by the relatively higher stability of Ho in solution, due to the formation of solution complexes with carbonate ligands, derived from the dissolution and hydration of volcanic CO_2_. The leaching process is accompanied by the simultaneous formation of Fe–Mn- oxohydroxides, testified by the almost ubiquitous negative Ce anomaly, due to the oxidative scavenging of Ce, occurring when the CO_2_-rich acidic waters interact with shallow and oxidizing zones of the aquifer.

## Data Availability

No datasets were generated or analysed during the current study.
